# Fortifier and Cream Improve Fat Delivery in Continuous Enteral Infant Feeding of Breast Milk

**DOI:** 10.3390/nu7021174

**Published:** 2015-02-11

**Authors:** Mika Tabata, Khaled Abdelrahman, Amy B. Hair, Keli M. Hawthorne, Zhensheng Chen, Steven A. Abrams

**Affiliations:** 1 Department of Bioengineering, Rice University, 6100 Main St., Houston, TX 77005, USA; 2 Department of Bioengineering, University of Pittsburgh, 4200 Fifth Ave., Pittsburgh, PA 15260, USA; E-Mail: kha9@pitt.edu; 3 Children’s Nutrition Research Center, Department of Agriculture/Agriculture Research Service, Department of Pediatrics, United States Baylor College of Medicine and Texas Children’s Hospital, 1100 Bates Ave., Houston, TX 77030, USA; E-Mails: abhair@bcm.edu (A.B.H.); kelih@bcm.edu (K.M.H.); zchen1@bcm.edu (Z.C.); sabrams@bcm.edu (S.A.A.)

**Keywords:** enteral nutrition, breast milk, human milk-derived fortifier, human milk-derived cream supplement, very low birth weight, neonates, Kangaroo ePump™, MedFusion™ pump, neonatal intensive care units

## Abstract

Premature and high-risk infants require accurate delivery of nutrients to promote appropriate growth. Continuous enteral feeding methods may result in significant fat and micronutrient loss. This study evaluated fat loss in enteral nutrition using current strategies for providing high-risk infants fortified human milk (HM). The fat content of HM was measured by IR analyzer in a simulated feeding system using the Kangaroo ePump™ and the MedFusion™ 2010 pump. Comparisons in fat loss were made between HM, HM supplemented with donor HM-derived fortifier Prolacta + H^2^MF™ (H^2^MF), and HM supplemented with H^2^MF and donor HM-derived cream ProlactCR™ (cream). When using the Kangaroo ePump™, the addition of H^2^MF and cream to HM increased fat delivery efficiency from 75.0% ± 1.2% to 83.7% ± 1.0% (*p* < 0.0001). When using the MedFusion™ 2010 pump, the addition of H^2^MF to HM increased fat delivery efficiency from 83.2% ± 2.8% to 88.8% ± 0.8% (*p* < 0.05), and the addition of H^2^MF and cream increased fat delivery efficiency to 92.0% ± 0.3% (*p* < 0.01). The addition of H^2^MF and cream to HM provides both the benefits of bioactive elements from mother’s milk and increased fat delivery, making the addition of H^2^MF and cream an appropriate method to improve infant weight gain.

## 1. Introduction

Very low birth weight (VLBW) and other high-risk infants require accurate delivery of nutrients in order to optimally grow during their hospital stay. The current feeding methods include syringe, bolus, and pump feeding. Human milk (HM) provides a wide range of benefits due to bioactive elements [1]. However, as previously shown [2,3,4,5,6], substantial amounts of fat and nutrients are lost throughout the delivery of HM by continuous syringe and pump feeding methods, due to fat adherence to the feeding bag, syringe and tubing. This fat adherence has been visually observed in previous studies [5]. Furthermore, limited assessment of the nutrients that reach the infant has been performed [7]. While minimal fat and nutrients from HM are lost through bolus feeding, many VLBW infants cannot tolerate the high flow rates required for bolus feeding. 

The Kangaroo ePump™ and MedFusion™ syringe pumps are prevalent in many neonatal intensive care units (NICUs), although a substantial amount of phosphorus, calcium, and other nutrients that bind to fat are lost in these delivery processes when used for continuous feedings [5]. When mother’s milk contains inadequate fat levels, many NICUs either replace HM with formula, which is not affected by fat adherence to tubing but does not contain bioactive elements found in HM, or add fortifiers such as HM-derived fortifier Prolacta + H^2^MF™ (H^2^MF) with or without donor HM-derived cream ProlactCR™ (cream) to HM to increase the fat content. ProlactCR™ cream is a pasteurized donor HM-derived fat from HM which yields 2.5 calories∙mL^−1^ and is intended as a supplement for mother’s own milk or donor HM in order to provide the infant with an exclusively HM-based diet. Evidence shows that an exclusively HM-based diet is associated with significantly lower rates of necrotizing enterocolitis (NEC) and surgical NEC when compared with preterm formula and with a mother’s milk-based diet supplemented with bovine milk–based products [8,9]. Furthermore, an exclusive HM-based diet is associated with lower mortality and morbidity in extremely preterm infants [10]. 

In order to improve the growth rate of premature and other high-risk infants, evaluations must be conducted to determine fat and nutrient losses due to different feeding methods and how it may be feasible to minimize these losses. The objective of this study was to evaluate fat loss in enteral nutrition using current strategies for delivering fortified HM to high-risk infants. We explore the use of HM-derived fortifier and cream to improve fat delivery efficiency, making it possible to deliver the bioactive elements of HM while increasing fat delivery to infants.

## 2. Experimental Section 

### 2.1. Human Milk Sample Preparation 

All HM used in this study was obtained through the mother’s milk bank of Texas Children’s Hospital in Houston, Texas and was designated for research purposes. HM from five anonymous mothers was pooled into a 4 L container, separated into aliquots in 50 mL VWR™ conical tubes, and stored in a freezer at −20°C. Before testing, HM was thawed with warm water and agitated using a common lab vortexer at 500 rpm to ensure homogeneity of the HM. No HM was used that had been thawed for over 24 h, which is the limit used by lactation services in the Texas Children’s Hospital NICU for feeding infants. 

HM with H^2^MF was prepared by mixing 20 mL of H^2^MF with 80 mL of HM, resulting in a 1:4 ratio of H^2^MF to HM. HM with H^2^MF and cream was prepared by combining 8 mL of cream with 20 mL of H^2^MF and 80 mL of HM, resulting in a 2:5:20 ratio of cream to H^2^MF to HM. These ratios follow clinical guidelines for HM containing 15–15.9 kcal∙oz^−1^ [11]. Although the average energy content of HM is approximately 20 kcal∙oz^−1^, there is great variability, and in the United States, values have been reported ranging from 13.8 kcal∙oz^−1^ to 25.1 kcal∙oz^−1^ with 51% of HM having less than 19.2 kcal∙oz^−1^ [12]. Fortification is recommended for HM containing less than 20 kcal∙oz^−1^ [11]. Thus, adding fortifier and cream in the ratio for HM containing 15–15.9 kcal∙oz^−1^ presumes the HM has a mid-range energy content of HM requiring fortification.

### 2.2. Experimental Setup/Feeding Simulations

All experiments in this study were conducted using a simulated continuous enteral feeding system, representing a typical NICU enteral feeding setup with variations between simulating feedings only in pump type and milk given. Variations of HM tested were HM, HM + H^2^MF, and HM + H^2^MF + cream. All simulated feedings used either the MedFusion 2010™ or the Kangaroo ePump™.

Before starting simulated feeds, 5 mL aliquots of HM were collected in 15 mL VWR™ tubes and processed for analysis of fat content directly thereafter, representing a “pre-feeding” aliquot.

The Kangaroo ePump™ was set up as it would be in typical NICU procedures. The bag (1000 mL Kangaroo ePump™ enteral pump set) was set 18 inches directly above the pump, and the collection site was level with the pump. The MedFusion™ pump was set up in the horizontal position and used with Monoject 35 mL syringes, which are made of polypropylene. Simulated feeds using the MedFusion™ pump were given through Covidien 60ES tubing extensions, which are made of polyvinyl chloride. Simulated feeds using the Kangaroo ePump™ were given through Kangaroo ePump™ 1000 mL Pump Sets (part #773656), which are made of polyvinyl chloride.

All simulated feeds were given at a flow rate of 20 mL∙h^−^^1^ for 60 min. Aliquots at 5, 10, 15, 30, 45, and 60 min after the start of the feed were collected in 15 mL VWR™ tubes and were processed for analysis of fat content directly after collection. 

### 2.3. Human Milk Sample Analysis

Prior to analysis, samples were warmed to 25–33 °C and homogenized using a probe sonicator (Q55 sonicator; Misonix Qsonica, Newton, Connecticut) for 10 s. HM samples were analyzed for fat concentration (g∙mL^−1^) and energy content (kcal∙oz^−1^) using a near-infrared milk analyzer (sample volume of 2 mL, Spectrastar™; Unity Scientific, Brookfield, Connecticut), following the Unity Scientific Procedure for Analyzing Breast Milk. For each milk sample, the average of three readings from the milk analyzer was recorded. The Spectrastar™ has been shown to measure fat content precisely but has room for improvement in accuracy [13].

### 2.4. Statistical Analysis

Total mass of fat as well as energy delivered throughout the entire duration of the simulated feed were calculated by right-handed integral approximation. The percentages of these approximations compared to the baseline fat content (“pre” feeding) integrated over the length of the feed represent the overall fat delivery efficiency. Data was analyzed using StatPlus® mac: LE (Copyright © 2010). Unpaired Student’s *t*-tests assuming unequal variances were performed to determine statistically significant differences between fat delivery efficiencies of these categories of HM. Statistical significance was reached if *p* < 0.05.

## 3. Results 

The addition of H^2^MF to HM affected overall fat delivery efficiency when the MedFusion™ pump was used but not when the Kangaroo ePump™ was used. When the Kangaroo ePump™ was used, simulated feeds with HM produced an overall fat delivery efficiency of 75.0 ± 1.2%, while simulated feeds with HM + H^2^MF produced an overall fat delivery efficiency of 75.9 ± 1.1% (Table 1). Thus, no significant difference in fat delivery efficiency was observed with the addition of H^2^MF to HM when using the Kangaroo ePump™ (*p* > 0.05). However, when the MedFusion™ pump was used, simulated feeds with HM produced an overall fat delivery efficiency of 83.2 ± 2.8%, while simulated feeds with HM + H^2^MF produced an overall fat delivery efficiency of 88.8 ± 0.8% (Table 1). Thus, a significant increase in fat delivery efficiency was observed with the addition of H^2^MF to HM when using the MedFusion™ pump (*p* < 0.05).

**Table 1:** Fat delivery efficiency of HM, HM + H^2^MF, and HM + H^2^MF + cream.

	HM	HM + H^2^MF	HM + H^2^MF + cream
Kangaroo ePump™	75.0% ± 1.2% (*n* = 8)	75.9% ± 1.1% (*n* = 8)	83.7 ± 1.0% (*n* = 8) *
MedFusion™	83.2% ± 2.8% (*n* = 9)	88.8% ± 0.8% (*n* = 6) *	92.0 ± 0.3% (*n* = 6) *

* Denotes statistical significance compared to corresponding simulated feed with HM; HM: human milk; H^2^MF: HM supplemented with donor HM-derived fortifier Prolacta + H^2^MF™; Cream: HM supplemented with H^2^MF and donor HM-derived cream ProlactCR™.

The addition of H^2^MF and cream to HM increased the overall fat delivery efficiency of both pumps. When the Kangaroo ePump™ was used, simulated feeds with HM + H^2^MF + cream resulted in an overall fat delivery efficiency of 83.7 ± 1.0%, compared to 75.0 ± 1.2% when HM alone was provided (*p* < 0.0001) and to 75.9 ± 1.1% when HM+H^2^MF was provided (*p* < 0.0001) (Table 1). When the MedFusion™ pump was used, simulated feeds with HM + H^2^MF + cream resulted in an overall fat delivery efficiency of 92.0 ± 0.3%, compared to 83.2 ± 2.8% when HM alone was provided (*p* < 0.01) and to 88.8 ± 0.8% when HM + H^2^MF was provided (*p* < 0.05) (Table 1). Therefore, when the MedFusion™ pump was used, the addition of cream further improved upon the fat delivery efficiency. When the Kangaroo ePump™ was used, an increase in fat delivery efficiency was observed only when both H^2^MF and cream were added to HM.

The average total fat (grams) and energy (kcal) delivered by the simulated one-hour feed increased with the addition of H^2^MF and further increased with the addition of cream (Table 2).

**Table 2:** Total fat/calories delivered over 20 mL, one-hour feeds (calculated with right-handed integral approximation).

	HM Fat (g)/Cal (kcal)	HM + H^2^MF Fat (g)/Cal (kcal)	HM + H^2^MF + cream Fat (g)/Cal (kcal)
Kangaroo ePump™	0.681/12.60	0.873/14.35	1.047/16.04
MedFusion™	0.765/13.55	0.986/16.01	1.178/17.91

HM: human milk; H^2^MF: HM supplemented with donor HM-derived fortifier Prolacta + H^2^MF™; Cream: HM supplemented with H^2^MF and donor HM-derived cream ProlactCR™.

**Figure 1:**
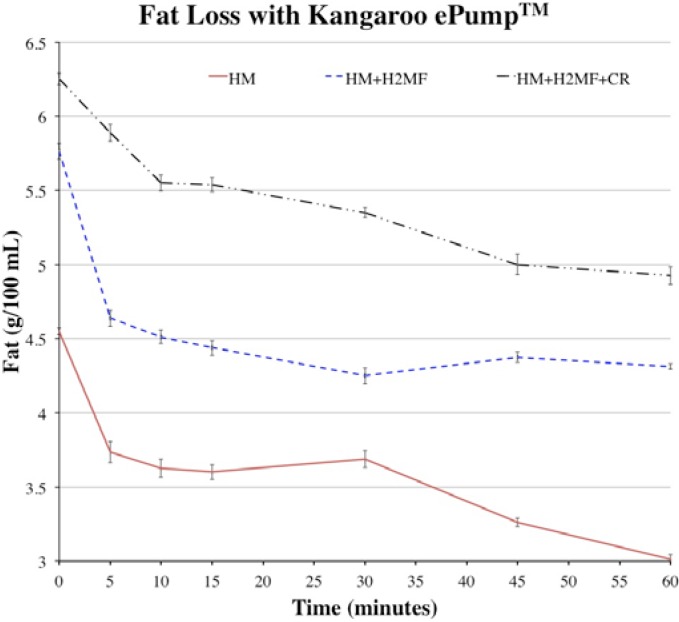
Fat values over one-hour feeds with HM, HM + H^2^MF, and HM + H^2^MF + cream with Kangaroo ePump™, HM: human milk; H^2^MF: HM supplemented with donor HM-derived fortifier Prolacta + H^2^MF™; Cream: HM supplemented with H^2^MF and donor HM-derived cream ProlactCR™.

**Figure 2:**
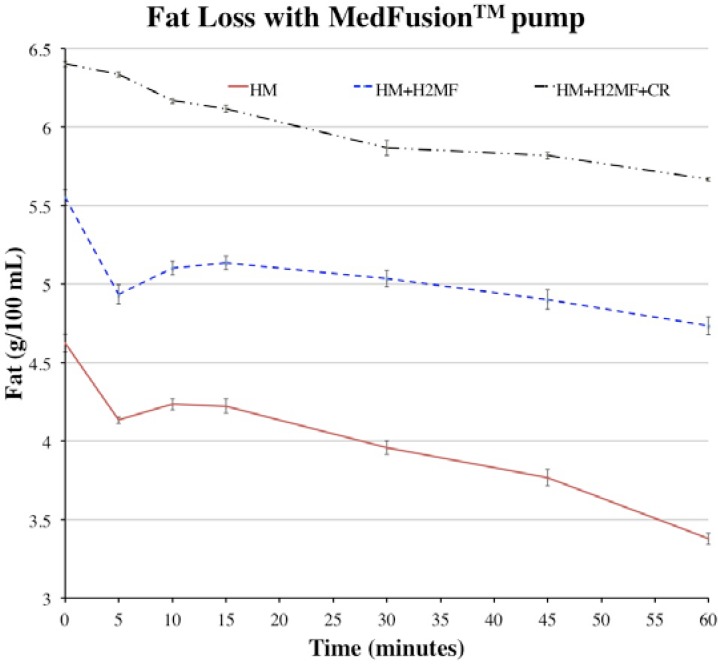
Fat values over one-hour feeds with HM, HM + H^2^MF, and HM + H^2^MF + cream with MedFusion™ syringe pump. HM: human milk; H^2^MF: HM supplemented with donor HM-derived fortifier Prolacta + H^2^MF™; Cream: HM supplemented with H^2^MF and donor HM-derived cream ProlactCR™.

The average fat values at each time point were plotted to show how fat content decreased throughout one-hour simulated feeds (Figure 1 and Figure 2). During simulated feeds using the Kangaroo ePump™, between *t* = 0 to 10 min, the fat content dropped by approximately half of the difference between the fat content at *t* = 0 min and *t* = 60 min (Figure 1). This revealed that a significant proportion of the total fat loss occurred in the first 10 min with all HM variations. By *t* = 60 min, the fat content of HM + H^2^MF and HM + H^2^MF + cream appeared to stop decreasing, but the fat content of HM continued to decrease after *t* = 60 min due to the negative slope seen in Figure 1. During simulated feeds using the MedFusion™ pump with HM and HM + H^2^MF, fat content dropped sharply between *t* = 0 to 5 min and then rose slightly between *t* = 5 to 10 min. After *t* = 15 min, fat levels gradually decreased. 

Lastly, all data supports the assertion that the MedFusion™ pump resulted in greater fat delivery than the Kangaroo ePump™, in agreement with previous studies. An increase was observed in overall fat delivery efficiency when using the MedFusion™ pump, compared to the Kangaroo ePump™, with each corresponding variation of HM (HM, HM + H^2^MF, and HM + H^2^MF + cream) (Table 1). Additionally, the MedFusion™ pump resulted in greater total fat and energy delivered in each variation of HM (Table 2).

## 4. Discussion 

Rogers *et al.* reported that the loss of fat, phosphorus, and calcium during continuous feeding pump methods is substantial, compared to the losses during gravity feeding [5]. Brooks *et al.* also showed a significant increase in fat loss with the MedFusion™ syringe pump compared to gravity feeding [4]. As the amount of fat lost over one-hour feeds is comparable to that lost during two-hour feeds, it appears that most of the fat loss occurs in the first hour. 

Few data are available regarding methodologies to decrease fat loss during continuous human milk feedings. Martinez *et al.* results indicate that fat separation can be prevented by ultrasonic homogenization of HM [14]. Furthermore, ultrasonic homogenization of HM appears to improve weight gain and triceps skin-fold thickness in premature neonates by minimizing fat loss, although further testing needs to be done on the safety of this technique [7]. Minibore tubing delivers more fat than standard bore tubing [2], although both types of tubing result in significant fat loss. Increased hydrophilicity of the feeding tube may help minimize clogging [15], and emulsifiers such as carrageenan may increase fat delivery but are currently limited by potential toxicity and lack of practical applicability to human milk feedings [16]. Supplementing enteral nutrition intakes of VLBW infants with H^2^MF and cream resulted in increased weight gain and length compared to the standard feeding regimen, supporting the use of H^2^MF and cream as a promising method to promote growth of infants [17].

We evaluated the addition of H^2^MF and cream as a potential practical method to increase fat delivery with HM. Results suggest that H^2^MF and cream can be used together to increase the fat delivery efficiency by almost 10% when using the Kangaroo ePump™ or the MedFusion™ syringe pump. The use of H^2^MF and cream increases fat delivery while maintaining the benefits of bioactive elements from HM, which may lead to improved weight gain and long-term outcomes. 

The calculations in Table 2 can be utilized to approximate the total fat and calories delivered by one-hour feeds at 20 mL·h^−1^ with the corresponding pump type, liquid type, and initial fat content (Figure 1 and Figure 2). The duration of the feed, the flow rate, and the initial fat content of milk affect overall fat delivery efficiency due to fat molecules occupying binding sites on the tubing and bag or syringe. Therefore, in order to accurately predict the total fat and calories delivered by feeds with various conditions, additional experiments must be performed with various feed duration, feed flow rate, and initial fat content. Calculations from these experiments could be utilized to more accurately track the fat that reaches the infant and to plan the appropriate mass of fat delivery in order to achieve proper infant growth rates. Excess fat consumption by infants, as well as insufficient fat consumption, can have negative impacts on childhood health, increasing the importance of tracking fat delivery [18].

Because the addition of H^2^MF and cream increases fat delivery efficiency, H^2^MF and cream appear to reduce the rate at which fat is lost to the tubing and bag or syringe. H^2^MF and cream were formulated through processes involving pasteurization [19], which induces changes in micelles [20], making it possible that H^2^MF and cream act as emulsifiers, preventing some of the fat from HM from separating out of the mixture. Alternatively, H^2^MF and cream may lose fat at a lower rate than HM loses fat, thereby increasing overall fat delivery efficiency without affecting the fat loss dynamics of HM. Lastly, a combination of these two possibilities may work to increase the overall fat delivery efficiency. 

When using the Kangaroo ePump™, significant fat loss in the first 10 minutes of feeds with all HM variations suggests that binding sites on the tubing and bag become saturated more quickly with fat during early stages of the feed. Later in the feed, the rate of fat loss decreases due to the decreased available binding sites. When using HM + H^2^MF and HM + H^2^MF + cream, which have high initial fat contents, these binding sites fill quickly, causing fat content to approach a lower limit by *t* = 60 min. Different trends of loss occur with the MedFusion™ pump than those with the Kangaroo ePump™, and these differences must be due to the differences between fat loss dynamics in the feeding bag versus the syringe because all other variables were held constant. 

Compared to the Kangaroo ePump™, when using the MedFusion™ pump, fat loss in the first 10 min is less rapid, and the overall trend of fat loss appears to be approximately linear. This may occur because the rate of fat molecule separation from the aqueous portion of milk is slower in a horizontal syringe than it is in a vertical feeding bag. The linear trend may be influenced by gradual, continuous fat separation in the syringe coupled with plunger movement. As fat separates toward the plunger end of the syringe, the plunger moves towards the syringe exit, pushing the fat in the same direction. Fat separation may explain the initial sharp drop in fat content, and plunger movement may explain the subsequent slight increase in fat content seen with HM and HM + H^2^MF from *t* = 5 to 10 min (Figure 2).

While this study suggests a practical method to increase fat delivery in enteral feeding with HM and provides insight into possible mechanisms of fat loss and retention, it contains the following limitations. Only one flow rate (20 mL·h^−1^) was tested, and it is known from previous studies that higher flow rates result in greater percent fat retained [7]. All simulated feeds were one hour in duration, but longer feeds will have decreased overall fat delivery efficiencies. Additionally, in a clinical setting, a nasogastric tube is attached to the end of the tubing used in this study, and additional fat loss may occur due to this extra tubing. However, to our knowledge, no studies have quantified the fat lost in nasogastric tubing, and this data can be used as a basis for further investigations designed to evaluate characteristics of fat loss from human milk-based diets.

## 5. Conclusions 

Fat and nutrient loss in continuous enteral feeding methods of HM remains a barrier to providing VLBW infants proper nutrients to grow and survive. Whenever possible, bolus-feeding methods should be utilized in lieu of syringe and pump methods with slow flow rates. However, for those infants who cannot tolerate bolus-feeding, this study proposes the addition of H^2^MF and/or cream to HM as a method to increase the percentage of fat delivered during infant enteral feeding. Our results suggests that the addition of H^2^MF + cream to HM is a practical method to increase fat delivery to VLBW infants while retaining the benefits of HM, which may lead to increased growth rates and improved long-term outcomes. Exploration of additional solutions to increase fat delivery efficiency at slow flow rates, particularly practical solutions, will provide further benefit for infants requiring enteral nutrition. 
